# Epidemiological patterns, temporal trends in management and long-term outcomes in testicular cancer: a 30-year single center experience

**DOI:** 10.1007/s12094-025-04068-9

**Published:** 2025-10-13

**Authors:** Patricia Capdevila, Jorge Aparicio Urtasun

**Affiliations:** Department of Medical Oncology, Hospital Universitario y Politécnico La Fe, Avda. Abril Martorell 106, 46016 Valencia, Spain

**Keywords:** Germ cell cancer, Testicular tumors, Prognosis, Chemotherapy

## Abstract

**Purpose:**

Recent studies have suggested a change in the epidemiologic pattern of testicular germ-cell tumors (TGCTs) and advances in therapeutic strategies have led to significant changes in their treatment over the last decades. Treatment guidelines for early-stage testicular cancer recommend de-escalation of therapy by the adoption of surveillance strategies. This study aimed to describe trends in TGCTs diagnosed over the last 30 years at our center and evaluate the impact of evolving treatment strategies.

**Methods:**

We retrospectively analyzed 277 TGCT patients treated from 1994 to 2023. Clinical characteristics, treatment patterns, and outcomes were assessed across three 10-year periods. Survival and relapse rates were estimated using Kaplan–Meier methods; significance was set at p < 0.05.

**Results:**

A significant increase in incidence was observed (p < 0.05), along more stage I diagnoses (p < 0.05) and older age at diagnosis, including a higher proportion aged ≥ 40 years (p < 0.001). Seminoma incidence doubled, while non-seminoma remained stable. In stage I disease, the use of active surveillance increased significantly, while adjuvant chemotherapy declined (p < 0.0005). Recurrence rates slightly decreased but were not statistically significant. Cause-specific 10-year survival improved from 95% to 97.2%, with overall survival stable at ~ 94%. The risk of second primary malignancies was notable, including contralateral testicular cancer (2.9%) and second malignant neoplasms (SMNs) (5.0%). A substantial burden of non-cancer-related causes of death were also observed.

**Conclusions:**

A real increase in the incidence of TGCTs and age at diagnosis was confirmed. These trends highlight a shift toward less aggressive treatment while maintaining excellent outcomes. However, the notable occurrence of SMNs and non-cancer mortality underscores the need for long-term follow-up that includes survivorship care beyond oncological monitoring.

## Introduction

Testicular germ cell tumors (TGCTs) account for approximately 1% of all male malignancies. In Eastern Europe and America, TGCTs are the most commonly diagnosed cancer among men aged 15 to 44 years [1]. Although historically increasing trends in Western Europe and worldwide [2–6] have started to stabilize in certain populations [7–10], others continue to experience rising incidence rates [11, 12]. A decline in incidence is predicted for countries like Italy and Spain [13]. Additionally, some studies suggest a reversal of the age trend with a shift toward older age at diagnosis [14–16].

This disease has become a model of curable neoplasm, mainly due to its exquisite sensitivity to cisplatin based combination chemotherapy and the development of a specialised multidisciplinary approach. Cure rates are now achievable for 95% of patients with testicular tumors, and 80% of those with metastatic disease. Despite its low mortality, optimizing management remains a key area of interest in oncology.

Over the past few decades, significant changes have occurred in the management of testicular cancer [17–20]. Current clinical studies are designed to tailor treatment as a function of individual patient risk, with the aim of minimizing the toxicity of therapy while maintaining its efficacy. For early-stage TGCTs, treatment guidelines have advocated de-escalation to avoid unnecessary interventions, and surveillance strategies have become increasingly adopted. Reports consistently show that these surveillance strategies result in excellent survival rates [21–25]. However, treatment patterns for stage I seminoma remain highly variable [26].

Despite therapeutic progress, real-world data describing long-term trends in the management and outcomes of TGCT across all stages are scarce. In particular, the clinical impact of evolving strategies—such as risk-adapted treatment and increased use of surveillance—remains insufficiently explored in large retrospective cohorts. To address this gap, we conducted a 30-year single-center study with the hypothesis that: (i) treatment intensity has declined following the adoption of individualized strategies; (ii) survival outcomes have remained stable; and (iii) long-term toxicities and second malignancies have become increasingly relevant.

This work aims to provide a comprehensive overview of temporal changes in TGCT epidemiology, management, and outcomes, contributing to the optimization of care and survivorship pathways.

## Materials and methods

A single-centre retrospective analysis of patients treated between January 1994 and December 2023 was conducted.

Data were extracted from 343 patients diagnosed with germ cell tumors from 1994 to 2023. A total of 18 patients were excluded from the study due to lack of information in medical records and 18 patients referred from another centre in a situation of recurrence for savage chemotherapy or transplantation. 31 patients presented with extragonadal disease. In total, 277 patients with TGCTs were analyzed.

Clinical and demographic data were extracted from medical records, including primary tumor location and histology, disease extent, serum tumor marker levels: beta-human chorionic gonadotropin (β-HCG), alpha-fetoprotein (AFP), and lactate dehydrogenase (LDH), history of testicular abnormalities, diagnostic methods, treatment details, response, follow-up period, and occurrence of metachronous testicular cancer or second malignancies. Follow-up duration and survival were calculated from the date of TGCT diagnosis until the last contact or death. Patients were classified into three consecutive 10-year periods (1994–2003, 2004–2013, and 2014–2023) to assess time trends. Histological features were reviewed locally. All clinical and pathological data were manually verified to ensure data integrity. Discordant or incomplete entries were cross-checked against the original medical records. Pathology reports were reviewed by experienced genitourinary pathologists at the time of diagnosis.

Continuous variables are presented as medians (interquartile range, IQR) and compared using the Wilcoxon rank-sum test. Group comparisons were conducted with analysis of variance (ANOVA) or the chi-squared test, as appropriate. Proportions were compared using Pearson’s χ^2^ or Fisher’s exact test. Overall survival (OS), cause-specific survival (CSS) and disease-free survival (DFS) were estimated using the Kaplan–Meier method, with comparisons performed via the log-rank test. Relapse rates were also reported as Kaplan–Meier estimates. Statistical significance was set at p < 0.05.

## Results

**Fig. 1:**
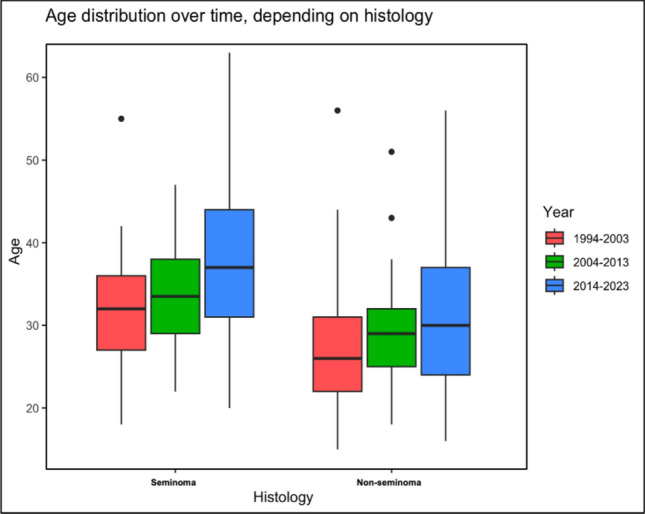
Comparison of distribution of age at diagnosis between the periods 1994–2003, 2004–2013 and 2014–2023, depending on histology. Box plots show the 25th and 75th percentiles (bottom and top of the box, respectively), median (band within the box), 2.5th and 97.5th percentiles (whisker terminals), and outliers

A total of 277 patients with TGCTs were evaluated, of which 169 (60.8%) were diagnosed at localized stages (stage I) and 107 (38.6%) at advanced stages (stages II and III). According to the IGCCCG classification, 36 patients (49.3%) were classified as having a good prognosis, 21 (28.8%) had an intermediate prognosis, and 16 (21.9%) had a poor prognosis. The median follow-up after orchiectomy was 119 months (range 5–362 months). The median age at diagnosis was 31 years (range 15–63), with seminoma patients having a median age of 35 years (range 18–63) and non-seminomatous germ cell tumor (NSGCT) patients a median age of 28 years (range 15–56). The median age at diagnosis progressively increased across periods: 29 years (range 15–56, n = 82) in period 1, 31 years (range 18–51, n = 83) in period 2, and 35 years (range 16–63, n = 112) in period 3, as depicted in Fig. 1.

Histology was influenced by the stage at diagnosis: 101 out of 169 patients (60.8%) in localized stages had seminoma, while 76 out of 107 patients (71.0%) in advanced stages had NSGCTs, with 21 of these 76 patients (27.6%) presenting embryonal carcinoma. Serum marker levels were approximately twice as high in advanced stages compared to localized stages (50.0% vs. 25.0%). Median serum marker values by stage are shown in Table 1. No significant differences were observed in tumor size between stages (< 40 mm vs. > 40 mm). However, lymphovascular and rete testis invasion were more frequent in advanced stages (48.2% vs. 31.3% and 25.6% vs. 19.8%, respectively). Table 2 summarizes the clinical and histologic characteristics of patients by stage. Among the 105 patients (37.8%) who presented metastases at diagnosis, 71 (67.6%) had retroperitoneal lymph node involvement, while 34 (32.4%) had either visceral metastases or lymph node involvement outside the retroperitoneum.

**Table 1:** Serum markers according to stage

	Stage	Median	Range	
BHCG	I	52	5	42,032
	II–III	143	5.8	228,370
AFP	I	161	8.8	8600
	II–III	253	9.5	44,235
LDH	I	445	330	3505
	II–III	590.5	335	13,369

**Table 2:** Clinical and histological characteristics according to stage

	Stage I	Stages II–III	*N* (%)
Global	169 (60.8)	107 (38.6)	277 (100)
Location			
Left	87 (51.4)	55 (51.4)	142 (51.3)
Right	80 (47.3)	50 (46.7)	130 (46.9)
Bilateral	2 (1.2)	1 (0.9)	3 (1)
Histology			
Seminoma	101 (60.8)	29 (27.3)	130 (47.8)
Non-seminoma	65 (39.2)	76 (71)	141 (50.9)
Histologic components			
Mixed tumor	55 (84.6)	44 (58.6)	99 (70.7)
Embryonic carcinoma	10 (15.4)	21 (27.6)	31 (22)
Yolk sac	0 (0)	4 (5.3)	4 (2.8)
Choriocarcinoma	0 (0)	4 (5.3)	4 (2.8)
Mature teratoma	0 (0)	2 (2.6)	2 (1.4)
Serum tumor markers			
Raised AFP	43 (26)	49 (47.6)	92 (34.3)
Raised BHCG	41 (25)	53 (52)	94 (35.3)
Raised LDH	70 (47.6)	62 (72.9)	132 (56.9)
Size			
< 40 mm	64 (44.4)	44 (48.9)	108 (46.1)
> 40 mm	80 (55.6)	46 (51.1)	126 (53.9)
Histological features			
Lymphovascular invasion	51 (31.3)	40 (48.2)	91 (37)
Rete testis invasion	32 (19.8)	21 (25.6)	53 (21.7)
In situ carcinoma	39 (24.4)	16 (19.3)	55 (22.4)
Testicular structures invasion			
No invasion	97 (59.1)	33 (39.8)	130 (52.6)
Albuginea	53 (32.3)	32 (38.5)	85 (34.4)
Epidídimo/vaginal	11 (6.7)	13 (15.7)	24 (9.7)
Spermal cord	3 (1.8)	5 (6)	8 (3.2)
IGCCCG classification			
Good prognosis	NA	36 (49.3)	36 (49.3)
Intermediate prognosis	NA	21 (28.8)	21 (28.8)
Poor prognosis	NA	16 (21.9)	16 (21.9)

**Fig. 2:**
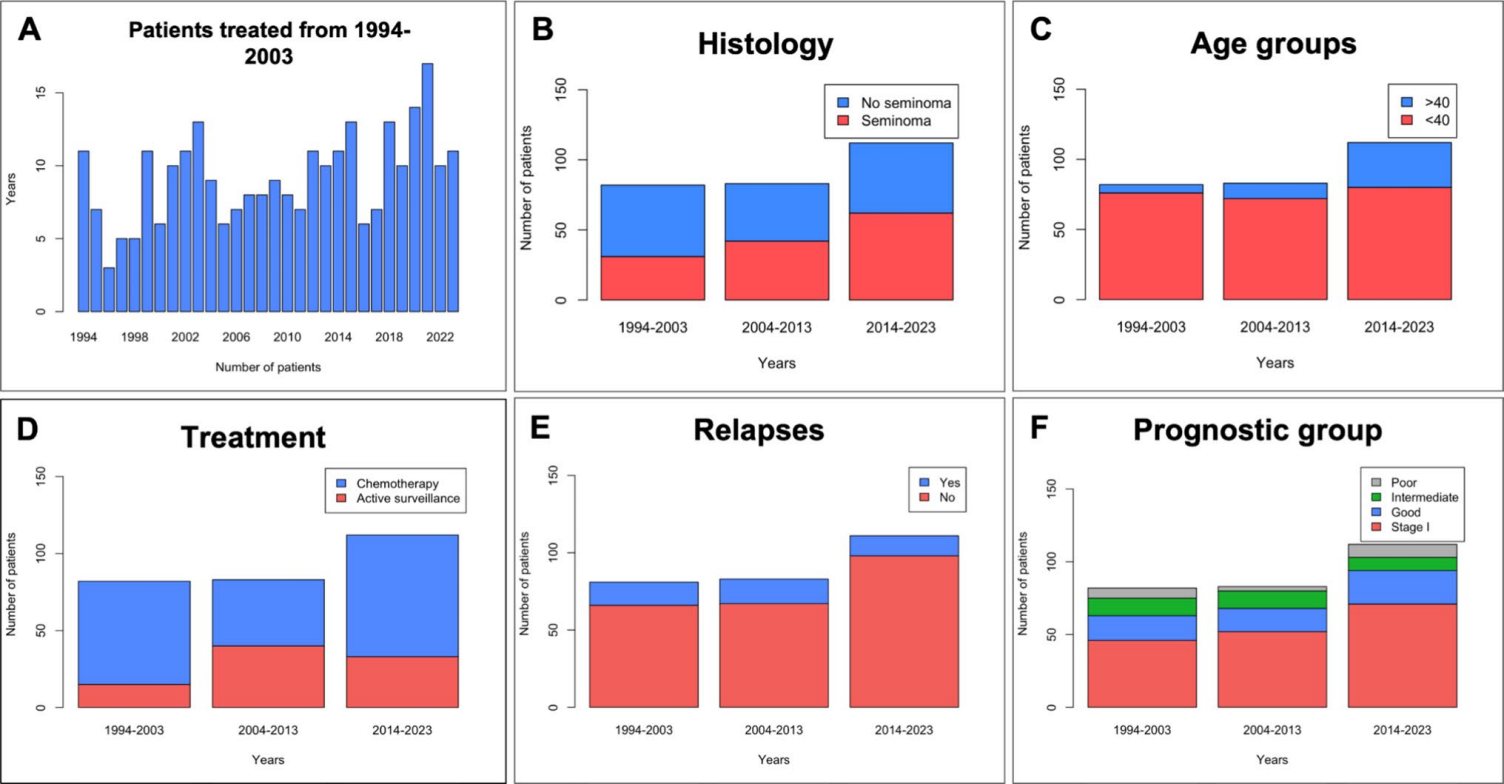
The number of patients with testicular GCTs treated over thirty years in a single spanish center (**A**). Comparison of pathological types (**B**), age at diagnosis (**C**), treatment (**D**), relapses (**E**) and prognostic group (**F**) in all patients between the periods 1994–2003, 2004–2013 and 2014–2023. Comparison of pathological types (**C**) and clinical stage (**F**) in patients aged ≥ 40 years between the periods 1994–2003, 2004–2013 and 2014–2023

**Fig. 3:**
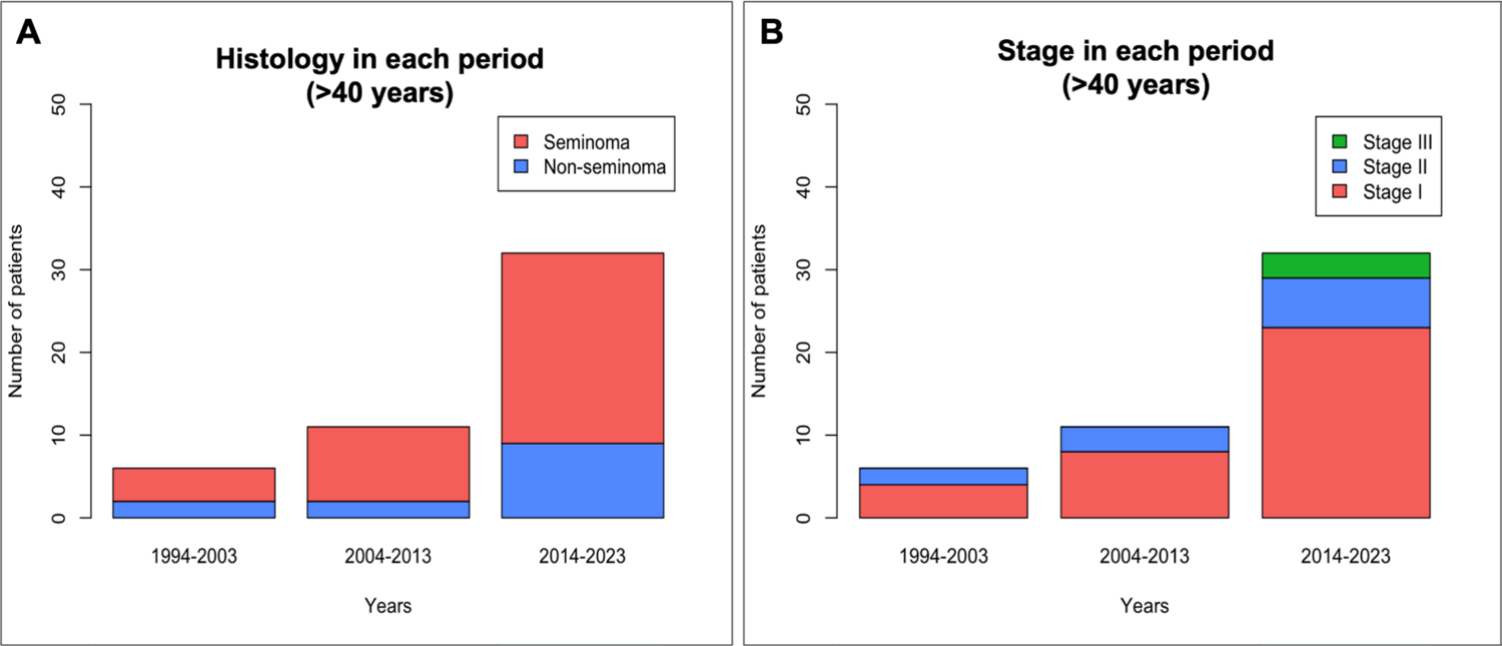
Comparison of pathological types (**A**) and clinical stage (**B**) in patients aged ≥ 40 years between the periods 1994–2003, 2004–2013 and 2014–2023

The incidence of TGCTs at our center increased over time, with 29.6% of cases diagnosed in the first decade (1994–2003), 30.0% in the second decade (2004–2013), and 40.0% in the third decade (2014–2023). Temporal trends are illustrated in Fig. 2. A progressive increase in the proportion of stage I cases was observed, along with a decline in stage II and III diagnoses over time. The relative incidence of stage I disease rose significantly, from 56.0% in 1994–2003 to 64.0% in 2013–2024 (p < 0.05). Among patients with metastatic disease, the proportion classified as IGCCCG good prognosis numerically increased. Histological trends revealed a twofold increase in the incidence of seminoma, with 31 cases (37.8%) diagnosed in 1994–2003 compared to 62 cases (55.4%) in 2013–2024 (p < 0.05). In contrast, the incidence of NSGCT remained stable, with 50 cases (44.6%) in the first period and 51 cases (62.2%) in the third. Age at diagnosis also increased significantly, with the proportion of patients aged ≥ 40 years rising from 7.3% in 1994–2003 to 13.3% in 2004–2013 and 28.6% in 2014–2023 (p < 0.001). Among those aged ≥ 40 years in the third period, seminoma was the predominant histology (71.8%), and most cases were diagnosed at stage I (71.8%), though small numbers preclude statistical value (Fig. 3). Rearding ethnicity, the incidence of TGCTs among Latin American patients increased over time, with 1 out of 82 cases diagnosed between 1994–2003, 8 out of 83 between 2004–2013, and 10 out of 102 between 2014–2023.

As expected based on our treatment policy, prophylactic retroperitoneal lymph node dissection (RPLND) and retroperitoneal irradiation have been replaced by adjuvant chemotherapy and active surveillance. The number of patients with stage I disease undergoing surveillance has increased, from 15 patients (32.6%) in the first period to 39 patients (75.0%) in the second period and 33 patients (46.4%) in the third period. Meanwhile, the proportion of patients receiving chemotherapy has significantly declined, from 81.7% in period 1 to 70.5% in period 3 (p < 0.0005). When analyzing recurrence rates over time in stage I patients, a non-significant trend was observed (p = 0.061, Chi-squared test), with a decrease in the most recent decade (11.1% in 1994–2003, 17.3% in 2004–2013, and 4.3% in 2014–2023). A Fisher’s exact test comparing periods 1 and 3 also did not reach statistical significance (p = 0.259).

Tables 3 and 4 summarize post-orchiectomy treatment strategies for early (Table 3) and advanced-stage disease (Table 4) based on histology and outcomes. Of the entire cohort, 223 patients (80.5%) remain in follow-up, 21 (7.6%) have died, and 33 (11.9%) are lost to follow-up.

**Table 3:** Treatment and outcomes in stage I GCT

	Seminoma (%)	Non-seminoma (%)	*N* (%)
Global	103 (61.3)	65 (38.7)	168 (100)
Initial treatment			
Active surveillance	51 (50.5)	34 (52.3)	85 (51.2)
Chemotherapy	46 (45.1)	31 (47.7)	77 (46.1)
Radiotherapy	3 (3)	0 (0)	3 (1.8)
RPLND	0 (0)	0 (0)	0 (0)
Chemotherapy schedule			
CBDCA	46 (100)	0 (0)	46 (61.3)
BEP	0 (0)	25 (86.2)	25 (33.3)
EP	0 (0)	4 (13.8)	4 (5.3)
Relapse			
Yes	12 (11.8)	5 (7.8)	17 (10.2)
No	90 (88.2)	59 (92.2)	149 (89.8)
Status at last follow-up			
Disease-free	100 (97)	60 (92.3)	160 (95.2)
Active disease	0 (0)	0 (0)	0 (0)
Died from cancer	0 (0)	0 (0)	0 (0)
Died from toxicity	0 (0)	1 (1.5)	1 (1.5)
Died other causes	3 (3)	4 (6.2)	7 (4.2)

**Table 4:** Treatment and outcomes in advanced GCTs (stages II and III)

	Seminoma (%)	Non-seminoma (%)	*N* (%)
Global	31 (29)	76 (71)	107 (100)
Initial treatment			
Chemotherapy	30 (96.8)	73 (98.6)	103 (98.1)
Radiotherapy	1 (3.4)	1 (1.4)	2 (0.2)
RPLND	4 (13.8)	18 (24)	22 (21.6)
Chemotherapy schedule			
CBDCA	1 (3.4)	0 (0)	1 (1)
BEP	9 (30)	38 (53.5)	47 (46.5)
EP	17 (56.6)	1 (1.4)	18 (17.8)
BEP-EP	3 (10)	24 (33.8)	27 (26.7)
BOMP-EPI	0 (0)	6 (8.5)	6 (5.9)
Others	0 (0)	2 (2.8)	2 (2)
Response			
CR	21 (67.7)	52 (69.3)	73 (68.9)
PR	9 (29)	15 (20)	24 (22.6)
No response	1 (3.3)	8 (10.7)	9 (8.5)
Relapse			
Yes	4 (12.9)	22 (29.7)	26 (24.8)
No	27 (87.1)	52 (70.3)	79 (75.2)
Status at last follow-up			
Disease-free	28 (90.3)	63 (82.9)	91 (85)
Active disease	1 (3.2)	1 (1.3)	2 (1.9)
Died from cancer	0 (0)	5 (6.6)	5 (4.7)
Died from toxicity	1 (0)	2 (2.6)	3 (2.8)
Died other causes	1 (3.2)	5 (6.6)	6 (5.6)

In stage I disease, 51.2% of patients were managed with active surveillance and 46.1% with adjuvant chemotherapy, regardless of histology. Among the 169 patients, 17 (10.2%) experienced relapse: 11 out of 102 patients with seminoma (10.8%) and 5 out of 65 with NSGCTs (7.7%). At the last follow-up in stage I, 160 patients (94.7%) were disease-free. One patient (0.6%) died due to treatment-related toxicity (septic shock after treatment administration), and 8 patients (4.7%) died from other causes: two suicides, one traffic accident, three from other malignancies (metastatic rhabdomyosarcoma, metastatic melanoma, and pancreatic cancer), one from liver cirrhosis, and one from cardiorespiratory arrest.

In advanced stages, 26 out of 107 patients (24.8%) experienced relapse. A total of 91 patients (85%) are disease-free, 2 (1.9%) have active disease, 5 patients (4.6%) have died from the tumor, and 3 (2.8%) from toxicity, including one case of neutropenic typhlitis and two septic shocks, one of which followed in-hospital pneumonia. Six patients (5.6%) died from other causes: three from different malignancies (prostate cancer and two colon cancers), one from SARS-CoV-2, one from terminal heart failure, and one from an unknown cause.

Regarding second malignancies, 8 patients (2.9%) developed a subsequent germ cell tumor (5 seminomas and 3 NSGCTs). Additionally, 14 patients (5%) presented with second cancers, including three colon cancers, one hepatocellular carcinoma, one Burkitt’s lymphoma, one acute myeloid leukemia (AML), one Hodgkin’s lymphoma, two melanomas, one meningioma, one mycosis fungoides, one prostate cancer, one rhabdomyosarcoma, and one patient who developed both pancreatic cancer and diffuse large B-cell lymphoma (DLBCL).

**Fig. 4:**
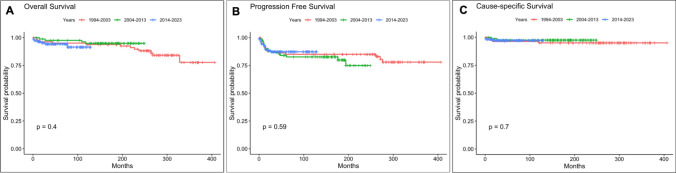
Comparison of overall survival (**A**), progression free survival (**B**) and cause-specific survival (**C**) among decades

The median follow-up for the cohort was 131 months (range: 1–370 months). CSS for localized stages at 5 and 10 years was 99.3%. In advanced stages (stages II and III), the 5-year CSS was 93.2%, and the 10-year CSS was 91.5%. The 5-year OS for localized stages was 96.7%, with a 10-year OS of 94.4%. For advanced stages, the 5-year OS was 93.2%, and the 10-year OS was 90.2%. When analyzed by periods, the 10-year OS was 93.8%, 94.9%, and 94.3% in periods 1, 2, and 3, respectively. The 10-year CSS was 95%, 97.5%, and 97.2% in those same periods. Figure 4 illustrates OS, CSS, and DFS across decades.

## Discussion

In this study, we describe temporal trends in practice and outcomes after orchiectomy for all cases of testicular cancer treated in our center between 1994–2023. Several important findings have emerged.

First, a progressive increase in the incidence of new cases (p < 0.05) and a higher frequency of stage I diagnoses (p < 0.05) were observed. This aligns with findings from previous large reports [2–6, 11, 12, 27], although in some countries, the increasing trend in incidence appears to have stabilized [7]. Advances in imaging and diagnostic protocols, together with greater clinical awareness, may have contributed to earlier diagnosis and more accurate staging. Regarding histology, our results show that the incidence of seminoma has doubled: 31 patients (37.8%) were diagnosed in 1994–2003 compared to 62 patients (55.4%) in 2013–2024 (p < 0.05). In contrast, the incidence of NSGCT remained stable, with 50 patients (44.6%) diagnosed in the first period and 51 patients (44.6%) in the latter. International trends also suggest a divergence in histological subtypes over the last decade, with seminoma rates increasing at a greater pace relative to NSGCTs [11]. These trends are consistent with those observed in previous reports [14, 16].

Second, an increase in the median age at diagnosis and in the proportion of patients aged ≥ 40 years was observed (28.6% in 2014–2023 compared to 7.3% in 1994–2003, p < 0.001). Previous studies have also suggested this age trend. Schaffar et al. (2019) in Switzerland did not report a significant increase of new cases, but the age-specific trends revealed that the most notable rise occurred in men aged 30–49 years [8]. Similar trends have been observed in Japan, with Yamashita et al. reporting that 28.8% of patients were aged > 40 years in the last decade, significantly higher than the 16.7% in the 1980 s [14]. Ozaki et al. found that the proportion of patients aged ≥ 40 years significantly increased in the period 2011–2021 (54.8%) compared to 2001–2010 (30.8%; p = 0.001) [15]. Additionally, Leveridge et al. (2018) reported that 42% of seminoma patients in Ontario were older than 40 years between 2000 and 2010 [18]. In contrast, in the Nordic countries, despite the overall increase in testicular cancer incidence, no significant differences were observed across age groups [9]. While the underlying reasons for these trends remain unclear, they may be partially explained by the aging population [28], although some studies suggest that lifestyle changes could also play a role [14, 15]. Regarding ethnicity, Almeida et al. reported that Latin American men have the highest TGCT incidence rates in the United States [29]. Our findings support this trend, with the proportion of Latin American men increasing from 1% in period 1 to 9.8% in period 3.

Regarding treatment over time, our results are consistent with the broader movement toward de-escalation of therapy, as reflected in treatment guidelines for early-stage TGCTs [17–25]. Specifically, there has been a marked increase in the use of surveillance for stage I cases, with 15/46 patients (32.6%) undergoing surveillance in period 1, 39/52 (75%) in period 2, and 33/71 patients (46.4%) in period 3. Conversely, the use of adjuvant chemotherapy has decreased, from 67.4% in period 1 to 53.5% in period 3. A recent systematic review showed long-term surveillance for clinical stage I germ cell tumors achieved comparable overall survival to adjuvant therapy, supporting surveillance as a valid strategy despite slightly increased relapse rates [30].

A large observational study also demonstrated a decrease in the use of adjuvant treatment in favor of observation for stage I seminoma, although this shift was not observed for NSGCTs [31]. Population data from Sweden and Norway showed a stable use of surveillance for stage I seminomas between 2000 and 2006, with rates ranging from 25 to 35% [32], similar to those in our study period. Leveridge et al. reported an increase in active surveillance rates among seminoma patients, rising from 56 to 84% between 2000 and 2010, while treatment for NSGCTs remained stable, with active surveillance rates ranging from 51 to 57% [18]. Weiner et al. observed an increase in surveillance for NSGCTs, from 65% in 2004–2005 to 74% in 2012–2013 [24]. While differences in surveillance rates across countries may be attributed to varying timelines for the implementation of surveillance policies, all results are consistent with an overall trend toward greater adoption of surveillance strategies.

Previous studies have demonstrated a decline in the use of RPLND [17–20] and radiotherapy as adjuvant treatments over time, a trend we confirm in our center. Notably, there have been no cases of RPLND for stage I since 1994, and only five patients received radiotherapy—three in localized stages and two in advanced stages.

When comparing recurrence rates over time, our study revealed a slight decrease despite the increased use of surveillance strategies. Among patients with stage I disease, 11.1% relapsed between 1994 and 2003, compared to 4.3% between 2014 and 2023. The Spanish Germ Cell Cancer Group study reported a 7% relapse rate in stage I seminoma with risk-adapted treatment from 2004 to 2008 [25]. A subsequent study in Spain, conducted between 1994 and 2015, showed a 7.8% relapse rate in 879 patients treated with risk-adapted approaches [33]. Mortensen et al. described a relapse rate of 18.9% (369/1954) in stage I seminoma patients on surveillance [21]. Our findings, with more recent data and extended follow-up, confirm that the shift toward treatment de-escalation through risk-adapted strategies does not compromise outcomes in routine practice, with no temporal changes in survival. These findings align with recent evidence from Boormans et al., who validated a three-factor model for predicting relapse in stage I seminoma. Incorporating such models into clinical decision-making may help optimize surveillance strategies and reduce overtreatment [34].

In our cohort, survival remained high, with 10-year cause-specific survival increasing from 95% in period 1 to 97.2% in period 3, while overall survival remained stable at approximately 94%.

The risk of a second primary cancer, including contralateral testicular cancer (2.9%) and second malignant neoplasms (SMNs) (5%), was significant and comparable to rates reported in other studies. The Spanish Germ Cell Cancer Group reported similar outcomes, with a 2% incidence of contralateral testicular cancer at 5 years and 4% at 14 years [35]. A recent population-based cohort reported a 15-year cumulative incidence of second malignant neoplasms (SMNs) of 6.7% (95% CI, 5.5–7.8), with the most common sites being digestive system cancers (1.9%), myeloid leukemias (1.1%), and cutaneous melanoma (0.8%). Contralateral testicular cancer occurred in 3.1% of survivors at 10 years [36].

Studies from other countries have shown comparable results. Wanderas et al. reported a 3.9% 15-year incidence in Norway [37], while Osterlind et al. observed a 5.2% 25-year incidence in Denmark [38]. Fossa et al. found a 1.9% 15-year incidence in the USA [39], and Akdogan et al. reported a 3% incidence in Turkey [40]. Similarly, Geczi et al. noted a 3% incidence in Hungary [41], and Hentrich et al. found a 4% incidence in Munich [42]. Andreassen et al. reported a 20-year incidence of 1.9% with cisplatin and 3.9% with pre-cisplatin treatment [43]. Rusner et al. described a standardized incidence ratio (SIR) in Germany ranging from 6 (3.3–10.1) to 13.9 (11.2–17) [44].

Compared to the general population, survivors of TGCTs experience a 1.7- to 3.5-fold increased overall risk of SMNs [45]. In the Surveillance, Epidemiology, and End Results (SEER) cohort, 2978 patients (5.6%) developed non-testicular cancer SMNs [46], which aligns with our results. While the increased risk of solid tumors in TGCTs survivors has primarily been attributed to radiotherapy, chemotherapy has also been linked to elevated risks [46]. Additionally, Fu et al. described testicular cancer patients who underwent radiation or chemotherapy had poorer SMN-specific survival [46]. Recently, concerns have risen about the increased risk of SMNs from radiation exposure during imaging surveillance. However, few studies have addressed this issue [47, 48], and the results remain inconclusive. At our center, surveillance protocols evolved over the three decades, progressively shifting toward less intensive imaging schedules. This included a reduction in the number of follow-up CT scans and, in more recent years, the omission of routine chest CTs in favor of chest X-rays or clinical evaluation in selected patients. Such changes may have contributed to minimizing cumulative radiation exposure and should be considered when interpreting long-term toxicity and the risk of second malignancies.

The overall risk of death in patients with testicular cancer remains significantly higher than in the general population. In our study, causes of death included four cases due to treatment-related toxicity (sepsis following chemotherapy), six from other malignancies, two from cardiovascular disease, two from suicide, one from cirrhosis, one from SARS-CoV-2 infection, and one from a traffic accident.

Fung et al. reported an increased mortality risk from pneumonia and influenza in TGCTs patients compared to the general population [49], while Fossa et al. observed a higher mortality risk due to fibrosis and pneumonia [50]. A recent U.S. population-based analysis identified accidents and adverse effects as the leading non-cancer cause of death (53 cases; 4.75%), followed by heart disease (45 cases; 4.04%), infectious diseases (27 cases; 2.42%), and vascular diseases (14 cases; 1.26%) [51]. However, beyond one year after diagnosis, heart disease became the most common non-cancer cause of death.

The incidence of suicide has also been reported to be higher among cancer patients [52], particularly in those with TGCTs [53], which aligns with our findings. Additionally, previous studies have documented an increased risk of sepsis following chemotherapy [51, 54], and our study further corroborates this, with deaths from sepsis occurring in TGCTs patients. These findings highlight the importance of long-term follow-up strategies that prioritize screening for secondary malignancies, cardiovascular diseases, treatment-related toxicities, and late complications [55]. Encouraging a healthy lifestyle, including smoking cessation, and adhering to the recommendations of the Exercise and Cancer Working Group of the Spanish Society of Medical Oncology (SEOM) would be advisable to help mitigate long-term health risks [56].

The present study has several limitations. As a retrospective database analysis from a single institution, it is subject to data quality concerns and inherent biases. When evaluating temporal trends, it is essential to acknowledge that selection bias cannot be excluded due to the referral area of our center. Moreover, the long study period (1994–2023) may introduce variability in diagnostic, therapeutic, and follow-up protocols, including changes in imaging technology, pathology reporting, and risk stratification criteria. Although we applied standardized definitions retrospectively to minimize inconsistencies, some heterogeneity may persist. The relatively small number of relapses, especially in subgroup analyses, might have limited statistical power to detect significant differences. Finally, the generalizability of our findings may be limited to similar centers with comparable patient populations and treatment strategies.

In summary, the incidence of testicular cancer has increased in recent years, primarily driven by a rise in stage I cases, seminomas, and diagnoses in patients over 40 years old. Concurrently, there has been a substantial de-escalation in treatment in routine practice since 1994, largely due to the increased adoption of surveillance strategies. This reduction in treatment intensity has not compromised survival. Our findings on SMNs and non-cancer-related mortality align with previously published studies and may help guide counseling on long-term health risks for men with testicular cancer.

## Data Availability

All data supporting the findings of this study may be available upon reasonable request and with prior authorization from Hospital Universitari i Politècnic La Fe and the Ethics Committee.

## Abbreviations

TGCT: Testicular germ cell tumor
NSGCT: Non-seminomatous germ cell tumor
RTI: Rete testis invasion
LVI: Lymphovascular invasion
OS: Overall survival
CSS: Cancer-specific survival
DFS: Disease-free survival
PFS: Progression-free survival
SMN: Second malignant neoplasm
RPLND: Retroperitoneal lymph node dissection
CBDCA: Carboplatin
BEP: Bleomycin, etoposide, and cisplatin
EP: Etoposide and cisplatin
BOMP-EPI: Bleomycin, oncovin, methotrexate, cisplatin/etoposide
AFP: Alpha-fetoprotein
β-HCG: Beta-human chorionic gonadotropin
LDH: Lactate dehydrogenase
IGCCCG: International germ cell cancer collaborative group
SEOM: Spanish society of medical oncology
SEER: Surveillance, epidemiology, and end results
